# Efficacy of vitamin D supplementation in reducing body mass index and lipid profile in healthy young adults in Colombia: a pilot randomised controlled clinical trial

**DOI:** 10.1017/jns.2022.108

**Published:** 2023-02-22

**Authors:** Norma C. Serrano, Lyda Z. Rojas, Edna M. Gamboa-Delgado, Diana Paola Suárez, Isail Salazar Acosta, Sandra L. Romero, Mario Forero, Doris C. Quintero-Lesmes

**Affiliations:** 1 Centro de Investigaciones, Fundación Cardiovascular de Colombia – FCV, Calle 155a No. 23–58, Floridablanca, Santander, Colombia; 2 Escuela de Nutrición y Dietética, Universidad Industrial de Santander, Cra. 32 No. 29-31, Bucaramanga, Santander, Colombia; 3 Escuela de Enfermería, Universidad Industrial de Santander, Cra. 32 No. 29-31, Bucaramanga, Santander, Colombia; 4 Neumología Pediátrica, Hospital Internacional de Colombia HIC – Instituto Cardiovascular, Fundación Cardiovascular de Colombia, Piedecuesta, Santander, Colombia

**Keywords:** Body mass index, Clinical controlled trial, Lipid profile, Vitamin D, Young adult

## Abstract

The objective of the present study was to evaluate the efficacy of oral administration of vitamin D supplementation in reducing BMI and lipid profile in adolescents and young adults from a cohort in Bucaramanga, Colombia. One hundred and one young adults were randomly assigned to one of two doses of vitamin D [1000 international units (IU) or 200 IU] administered daily for 15 weeks. The primary outcomes were serum 25(OH)D levels, BMI and lipid profile. The secondary outcomes were waist-hip ratio, skinfolds and fasting blood glucose. We found a mean ± sd plasma concentration of 25-hydroxyvitamin D [25(OH)D] was 25⋅0 ± 7⋅0 ng/ml at baseline, and after 15 weeks, it increased to 31⋅0 ± 10⋅0 ng/ml in the participants who received a daily dose of 1000 IU, (*P* < 0⋅0001). For the participants in the control group (200 IU), it went from 26⋅0 ± 8⋅0 ng/ml to 29⋅0 ± 8⋅0 ng/ml (*P* = 0⋅002). There were no differences between groups in body mass index. There was a statistically significant decrease in LDL-cholesterol between the intervention group *v.* the control group (mean difference −11⋅50 mg/dl (95 % CI −21⋅86 to −1⋅15; *P* = 0⋅030). The conclusions of the present study were two different doses of vitamin D supplementation (200 IU *v*. 1000 IU) produced changes in serum 25(OH)D levels over 15 weeks of administration in healthy young adults. No significant changes were found in the body mass index when the effect of the treatments was compared. A significant reduction in LDL-cholesterol was found when comparing the two intervention groups.

Trial registration: NCT04377386

## Introduction

Vitamin D deficiency constitutes a worldwide public health problem. It is highly prevalent even in countries such as Colombia with a hot tropical climate^(1)^, where it is deemed that there is sufficient ultraviolet (UV) radiation throughout the year, or in developed countries where vitamin D fortification has been implemented^(2)^. The prevalence of vitamin D deficiency in young adults ranges from 20 to 80 %. In two populations of Colombian young adults, it was estimated to be 22⋅4 %^(3)^. Whereas, it was reported to be 27 % in Chile, 28 % in Mexico, 42 % in the United States and even 77 % in Brazil^(3,4)^.

Serum vitamin D deficiency has been associated with several chronic diseases, including cardiovascular disease, stroke and diabetes^(5–7)^. Likewise, low vitamin D serum levels have been reported in obese people compared with those of normal weight^(8)^. Controlled clinical trials with vitamin D supplements have shown an inverse response in the concentration of 25(OH)D according to the state of the body mass index (BMI) and body fat, where the lower levels of 25(OH)D are observed in obese and overweight people. It is probably because vitamin D is distributed in a larger body volume or because of a slower release into the circulation of that stored in adipose tissue^(9)^. Another study reported that after vitamin D supplementation, all obese women reach adequate levels of 25(OH)D in serum, but normal women (BMI < 25 kg/m^2^) achieve higher levels, so in this group, lower doses of vitamin D should be used^(10)^. Moreover, a meta-analysis quantifying the effects of vitamin D supplementation indicated that obesity indices did not improve significantly despite increased serum 25(OH)D levels^(11)^.

Vitamin D deficiency has also been associated with dyslipidemia, although the evidence is conflicted^(12)^. Some studies carried out in normal weight and obese adults from China, Hawaii and Mexico have shown favourable lipid profiles in those individuals supplemented with vitamin D. In these, exposed participants received for 6 months a daily vitamin D dose of 2000, 4000 and 800 IU, respectively^(13–15)^. However, others have also found unfavourable results for high-density lipoprotein (HDL)-cholesterol after vitamin D supplementation in adult populations of colorectal adenoma patients and postmenopausal women from the United States. The implemented doses were 800 IU daily for 6 months and 400 IU daily for 4 years, respectively^(16,17)^. Meanwhile, other intervention studies have documented favourable results but with insignificant effects of vitamin D on serum lipid profiles in postmenopausal women from Malaysia and overweight adults from Germany. The implemented doses were 50 000 IU weekly for 2 months and then once a month for 10 months, and 3332 IU daily for 12 months, respectively^(18,19)^.

The exact mechanisms by which low vitamin D levels occur in people with obesity and influence the lipid profile have not been fully elucidated^(20–22)^. Likewise, the results of interventional studies evaluating the effect of vitamin D supplementation on excess weight (overweight/obesity) and serum lipid profile in relatively healthy people at an early age remain uncertain and inconsistent. The heterogeneity among studies can be attributed mainly to the duration of vitamin D supplementation and the baseline lipid profile^(4,22)^. Nevertheless, vitamin D supplementation has been suggested to alleviate metabolic syndrome, specifically its atherogenic dyslipidemia component^(22)^.

These associations are of clinical relevance and its study carries important public health implications related to the possible benefit from vitamin D supplementation in cardiovascular and metabolic health conditions^(22,23)^. Additionally, there are few intervention studies in the Latin American population, in which seasonal variation does not have such a marked effect on vitamin D levels. Therefore, the present study was conducted with the objective of evaluating the efficacy of oral administration of vitamin D supplementation in reducing BMI and lipid profile in adolescents and young adults from a cohort in Bucaramanga, Colombia.

## Materials and methods

### Design

**Fig. 1.**
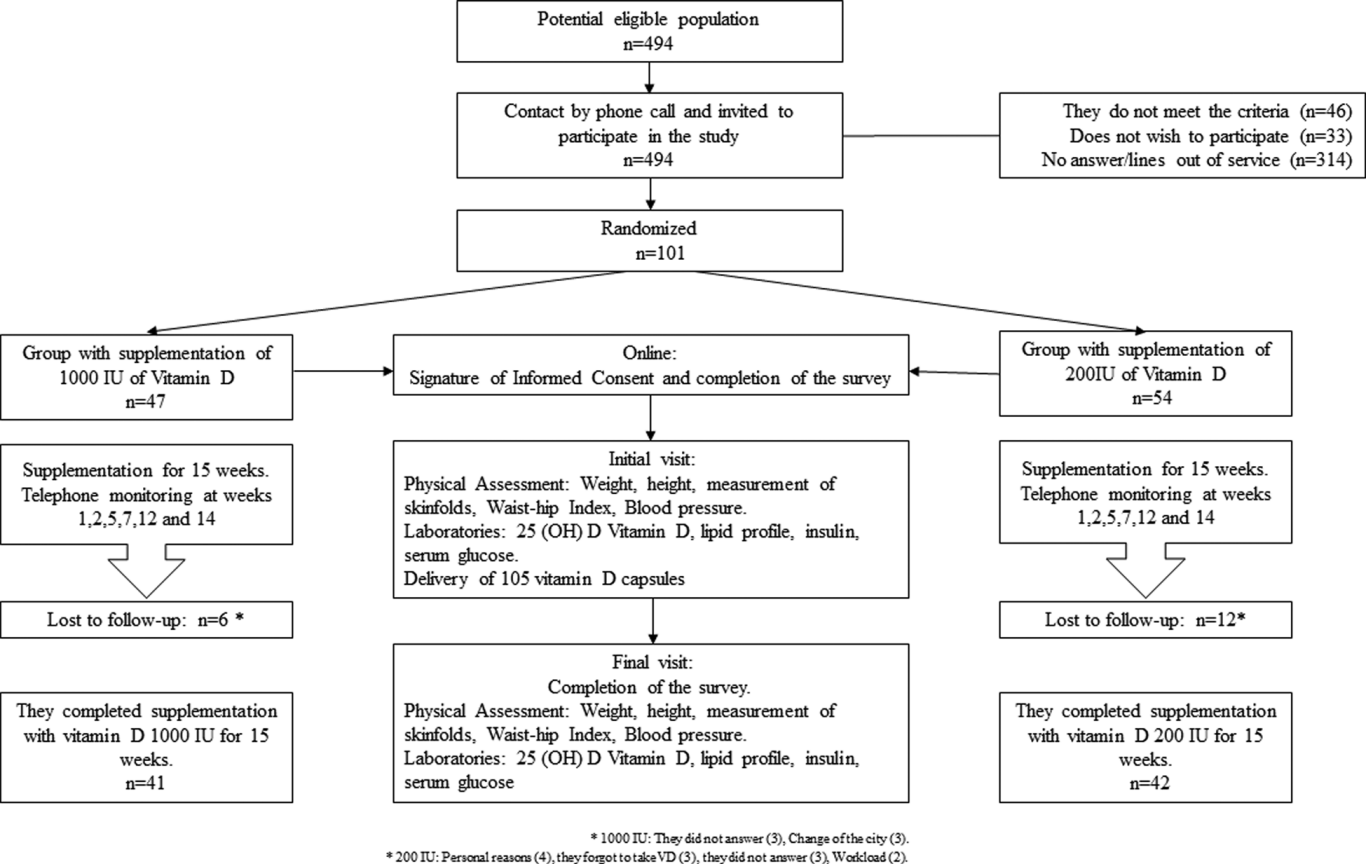
Follow-up of the participants throughout the intervention.

A triple-blind parallel two-arm randomised controlled clinical trial. The study participants were young adults from the SIMBA cohort, which began in 2006 with 1282 children between 6 and 10 years of age in the city of Bucaramanga, Colombia (latitude: 7⋅12539)^(24,25)^. The study was carried out between the months of July and December 2021. In Bucaramanga, there are no seasons. Between 2013 and 2017, the SIMBA II project was carried out, which managed to establish contact and follow-up with 494 adolescents^(26)^. Finally, in 2019, 217 participants were contacted by phone call. The latter constituted the eligible population for this study (Fig. 1).

**Fig. 2.**
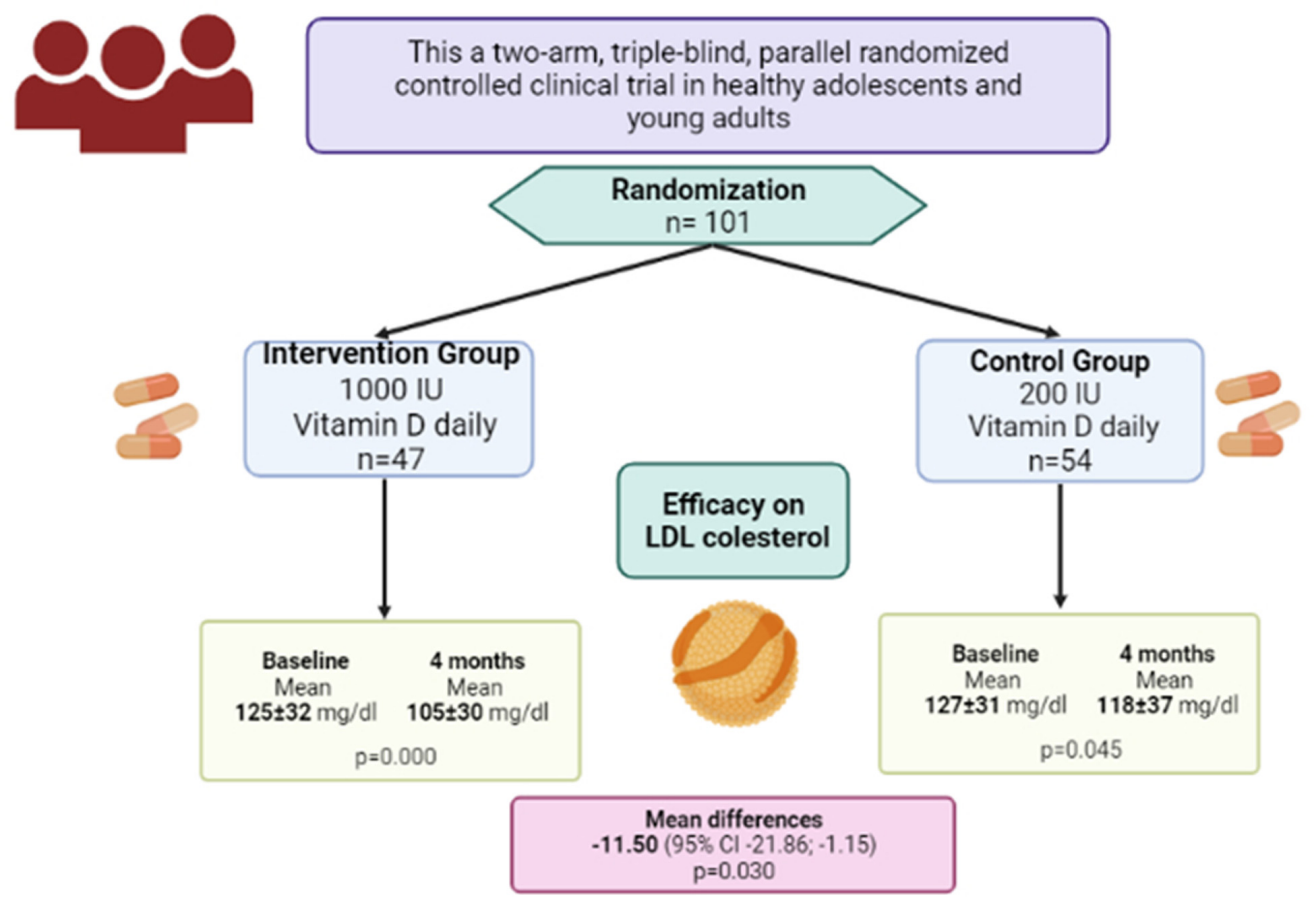
Representation of the main finding of this study.

The protocol was registered in Clinicaltrials.gov with the NCT04377386 registry, and all methodological aspects related to the study have been previously published^(27)^ (Fig. 2).

### Intervention

The intervention group received 1000 IU of vitamin D and the control group 200 IU of vitamin D daily for 15 weeks. All participants were given 105 doses of vitamin D in white containers identical in terms of organoleptic characteristics to ensure that both participants and researchers were blinded to treatment. The supplementation was provided by the Farma de Colombia laboratory, commercially known as Farma D, whose presentation is in soft gelatin capsules of vitamin D3. To maximise adherence to the intervention, the participants crossed off the daily intake of the supplement on a calendar with the period of the study, which they returned at the end of it. On the other hand, all participants were followed up by phone call at weeks 1, 2, 5, 7, 12 and 14; in these calls, they were inquired about any ‘potential’ adverse effect related to the intervention and adherence to taking vitamin D was evaluated through a self-report instrument^(27)^.

The cut-off points to establish vitamin D levels were: Deficiency (≤20 ng/ml); Insufficiency (21–29 ng/ml) and Sufficiency (≥30 ng/ml)^(28)^.

### Sample size

It was calculated taking into account an expected difference in the results of the study (overweight, obesity and altered lipid profile) of 20 %^(29)^ between the intervention group and the control group, a power of 90 %, an alpha of 5 %, a group relationship intervention/control group ratio of 1:1 and an adjustment of 20 % for losses to follow-up, resulting in a sample of 270 participants (*n* 135 intervention group and *n* 135 control group); for the calculation, the OpenEpi program was used. On account, it is a pilot study and considering the people who were able to be re-contacted by telephone, the sample size achieved was 101 participants (*n* 41 intervention group and *n* 42 control group).

### Outcomes

The primary outcomes were serum 25(OH)D levels, BMI and lipid profile. The secondary were waist-hip ratio, skinfolds and fasting blood glucose.

### Statistical methods

An intention-to-treat analysis was performed. The description of the categorical variables was made using absolute and relative values; of the quantitative variables that presented a normal distribution in the Shapiro–Wilk test the mean and standard deviation were reported, otherwise, the median and interquartile range [IQR] were presented. The baseline characteristics of the study groups (intervention and control) were compared using the proportions comparison test (χ^2^ or Fisher's exact) and Student's *t* test or Mann–Whitney *U* test, as appropriate.

The outcome variables were analysed quantitatively and contrasted through paired *t* tests. In addition, the mean difference was calculated for each of the results, which were analysed through linear regressions where the principal exposure was the treatment [intervention (1000 IU of vitamin D) *v.* the control group (200 IU of vitamin D)] adjusted by the initial value of the outcome. Total energy per intake and triacylglycerols were not balanced at the beginning of the study. However, they were not considered confounding factors for the relationship being studied, therefore, they were not adjusted. Statistical significance was considered for all hypothesis tests when the *P*-value was less than 0⋅05. All data were analysed in Stata statistical software, version 15.0 (Stata Corporation, College Station, TX, USA).

### Ethical considerations

This study was conducted according to the guidelines laid down in the Declaration of Helsinki and all procedures involving human subjects/patients were approved by the Research Ethics Committee of the Fundación Cardiovascular de Colombia (Record No. 480 of July 16, 2019). Written informed consent was obtained from all subjects.

## Results

The potentially eligible population sample was 494 adolescents and young adults (Fig. 1). When contacted to participate in the study, 46 did not meet the inclusion criteria, 33 did not wish to participate and 314 did not answer or their telephone was out of service. Therefore, 101 participants met the eligibility criteria for the study and were randomly assigned to one of two treatment arms: 47 individuals in the 1000 IU/d group and 54 individuals in the 200 IU/d group. At the end of the 15-week follow-up (4 months), 41 (87⋅2 %) and 42 (77⋅7 %) completed the study, respectively. A comparison of the main socio-demographic and clinical variables related to the study topic was made, and no statistically significant differences were found between the participants who completed the follow-up and those who were lost (*P* > 0⋅05).

### Baseline characteristics of study participants

The average age of the participants was 22⋅6 [±1⋅5] years, 56⋅44 % were women, with a median BMI of 23⋅3 [Q1: 20⋅8; Q3: 26⋅8]. Median baseline serum vitamin D [25(OH)D] levels for the 1000 IU group were 23⋅3 ng/ml [Q1: 20⋅2; Q3: 31⋅3], and for the 200 IU of 24⋅7 ng/ml [Q1: 21⋅6; Q3: 31⋅6]. No differences were found between groups concerning baseline characteristics, except for total energy intake and serum triacylglycerol levels. The baseline characteristics of the participants according to the treatment group are shown in Table 1.

**Table 1.** Baseline characteristic of participants by vitamin D intervention group (*n* 101)

Variables	All	Intervention group 1000 IU (*n* 47)	Control group 200 IU (*n* 54)	*P*-value
Sex, *n* (%)				
Women	57 (56·44)	27 (57·45)	30 (55·56)	0·848
Men	44 (43·56)	20 (42·55)	24 (44·44)	
Age (years), mean [sd]	22·6 [±1·5]	22·6 [±1·5]	22·7 [±1·6]	0·837
City of residence, *n* (%)				
Bucaramanga	52 (51·49)	26 (55·32)	26 (48·15)	0·717
Others (Floridablanca, Barrancabermeja, Girón, Piedecuesta)	8 (7·92)	4 (8·51)	4 (7·41)	
No data	41 (40·59)	17 (36·17)	24 (44·44)	
Socioeconomic status, *n* (%)				
Low	55 (54·46)	22 (46·81)	33 (61·11)	0·150
Medium	46 (45·54)	25 (53·19)	21 (38·89)	
Marital status, *n* (%)				
Single	87 (86·14)	41 (87·23)	46 (85·19)	0·883
Married or free union	12 (11·88)	5 (10·64)	7 (12·96)	
Separated, divorced, widowed	2 (1·98)	1 (2·13)	1 (1·85)	
Number of children, *n* (%)				
0	83 (82·18)	42 (89·36)	41 (75·93)	0·253
1	15 (14·85)	4 (8·51)	11 (20·37)	
2	3 (2·97)	1 (2·13)	2 (3·70)	
Currently working, *n* (%)	49 (48·51)	22 (46·81)	27 (50·00)	0·749
Currently studying, *n* (%)	52 (51·49)	24 (51·06)	28 (51·85)	0·937
Education, *n* (%)				
Doesn't have a high school	1 (0·99)	1 (2·13)	0 (0·00)	0·558
High school	24 (23·76)	13 (27·66)	11 (20·37)	
Incomplete high school	5 (4·95)	3 (6·38)	2 (3·70)	
Complete technician	20 (19·80)	8 (17·02)	12 (22·22)	
Incomplete university	35 (34·65)	13 (27·66)	22 (40·74)	
University	14 (13·86)	7 (14·89)	7 (12·96)	
Incomplete postgraduate	1 (0·99)	1 (2·13)	0 (0·00)	
Postgraduate	1 (0·99)	1 (2·13)	0 (0·00)	
Sun exposure at 10:00 a.m. and 4:00 p.m. Yes, *n* (%)	51 (50·50)	26 (55·32)	25 (46·30)	0·366
Total physical activity (meets), median [Q1; Q3]	720 [99; 3375]	1188 [198; 4186]	693 [96; 3375]	0·425
Tobacco and alcohol consumption				
Ever smoked in your life, *n* (%)	28 (27·72)	15 (31·91)	13 (24·07)	0·380
Currently smoke, *n* (%)	4 (14·29)	2 (13·33)	2 (15·38)	1·000
Alcohol consumption frequency, *n* (%)	66 (65·35)	33 (70·21)	33 (61·11)	0·338
Dietary intake				
Energy (kcal/d)	2002 (1663; 2212)	1887 (1574; 2010)	2008 (1782; 2373)	**0·046**
Calcium (mg/d)	839 (820; 882)	839 (811; 888)	841 (821; 876)	0·518
Vitamin D (μg/d)	0·4 (0·2; 0·6)	0·4 (0·2; 0·6)	0·4 (0·3; 0·6)	0·315
Vital signs				
Systolic blood pressure (mmHg), mean [sd]	114 [±12]	114 [±13]	114 [±12]	0·913
Diastolic blood pressure (mmHg), mean [sd]	66 [±8]	66 [±7]	66 [±8]	0·725
Heart rate (lpm), mean [sd]	69 [±10]	70 [±11]	69 [±9]	0·683
Skin folds				
Bicipital (mm), median [Q1; Q3]	10 [6; 12]	10 [7; 13]	10 [5; 12]	0·422
Sub-scapular (mm), median [Q1; Q3]	15 [12; 21]	15 [12; 21]	15 [12; 21]	0·832
Tricipital (mm), median [Q1; Q3]	17 [12; 22]	17 [14; 22]	16 [11; 23]	0·447
Abdominal (mm), median [Q1; Q3]	20 [15; 25]	20 [17; 24]	20 [14; 25]	0·738
Anthropometry				
Body mass index, median [Q1; Q3]	23·3 [20·8; 26·8]	23·3 [21·0; 27·1]	23·4 [20·6; 26·4]	0·504
Waist-hip ratio, median [Q1; Q3]	0·79 [0·74; 0·83]	0·78 [0·74; 0·84]	0·79 [0·74; 0·83]	0·764
Serum biomarkers				
Total cholesterol (mg/dl), median [Q1; Q3]	181 [161; 200]	171 [157; 200]	185 [168; 206]	0·164
HDL-cholesterol (mg/dl), median [Q1; Q3]	41 [37; 47]	40 [36; 47]	41 [38; 48]	0·286
LDL-cholesterol (mg/dl), median [Q1; Q3]	117 [104; 136]	115 [99; 136]	119 [105; 139]	0·687
Triacylglycerols (mg/dl), median [Q1; Q3]	90 [66; 118]	83 [65; 98]	96 [70; 121]	**0·049**
Glucose (mg/dl), mean [sd]	88 [±6·6]	87·7 [±6·8]	87·7 [±6·4]	0·986
Insulin (μU/ml), median [Q1; Q3]	7·4 [5·1; 9·4]	7·3 [5·4; 8·9]	7·6 [4·9; 9·8]	0·886
HOMA-IR	1·6 [1·1; 2·1]	1·6 [1·1; 1·9]	1·6 [1·1; 2·1]	0·915
Vitamin D (ng/ml), median [Q1; Q3]	23·9 [20·8; 31·3]	23·3 [20·2; 31·3]	24·7 [21·6; 31·6]	0·489

Q, quartile; sd, standard deviation; HOMA-IR, homeostatic model assessment for insulin resistance. p values with statistical significance.

### Effect of vitamin D supplementation

Treatment adherence was evaluated at the end of the study for each of the groups with the count of tablets. The median [Q1; Q3] of this was 102 [98; 105] for the 1000 IU group and 99·5 [92; 102] for those who received 200 IU, without statistically significant differences (*P* = 0·453). Additionally, no side effects were reported in any of the intervention groups.

A significant reduction in the values of total cholesterol, HDL and glucose was observed in both groups, separately. When compared between groups, these variables lacked statistical significance. On the other hand, there was a significant reduction of triacylglycerols (mean 105 *v*. 91 mg/dl, *P* = 0·045) in the intervention group, while the change in the control group was slight. The BMI increased slightly in the intervention group (0·2 [0·0] kg/m^2^), being statistically significant (*P* = 0·026), while there was no change in the control group.

Regarding the treatment comparison, a statistically significant decrease in LDL-cholesterol and triacylglycerol levels were observed when evaluating the intervention group *v*. the control group (mean difference −11·92 mg/dl [95 % CI −21·98 to −1·85; *P* = 0·021]) and group (mean difference −15·14 mg/dl [95 % CI −21·29 to −0·98; *P* = 0·036]). Also, a statistically significant increase in vitamin D levels was observed when comparing the intervention group *v.* the control group (mean difference of 3·72 IU [95 % CI 0·93 to 6·50; *P* = 0·009]). The effect of the dose of vitamin D supplementation during 4 months of follow-up can be seen in Table 2.

**Table 2.** Effect of the intervention (vitamin D supplementation) on outcomes during 4 months follow-up (*n* 83)

Variables	Intervention group 1000 IU (*n* 41)			Control group 200 IU (*n* 42)			Treatment comparison	
	Baseline	4 months	*P*-value*	Baseline	4 months	*P*-value*	Mean differences (95 % CI)	*P*-value**
Primary outcomes								
Body mass index, mean [sd]	25·1 [±5·6]	25·3 [±5·6]	**0·026**	23·6 [±4·3]	23·6 [±4·4]	0·939	0·01 (−0·01; 0·02)	0·308
Total cholesterol (mg/dl), mean [sd]	185 [±38]	177 [±31]	**0·014**	192 [±35]	186 [±37]	**0·023**	−3·38 (−10·69; 3·93)	0·360
HDL-cholesterol (mg/dl), mean [sd]	41 [±8]	48 [±10]	**0·000**	43 [±9]	48 [±9]	**0·000**	1·72 (−0·86; 4·30)	0·189
LDL-cholesterol (mg/dl), mean [sd]	125 [±32]	105 [±30]	**0·000**	127 [±31]	118 [±37]	**0·045**	−11·92 (−21·98; −1·85)	**0·021**
Triacylglycerols (mg/dl), mean [sd]	105 [±74]	91 [±56]	**0·045**	103 [±43]	104 [±48]	0·763	−15·14 (−29·29; −0·98)	**0·036**
25(OH)D (ng/ml), mean [sd]	25 [±7]	31 [±10]	**0·000**	26 [±8]	29 [±8]	**0·002**	3·72 (0·93; 6·50)	**0·009**
Secondary outcomes								
Waist-hip ratio, mean [sd]	0·79 [±0·07]	0·79 [±0·07]	0·844	0·79 [±0·05]	0·78 [±0·05]	0·157	0·01 (−0·01; 0·02)	0·301
Bicipital skinfold (mm), mean [sd]	10·6 [±5·4]	10·5 [±5·4]	0·912	9·7 [±5·6]	9·7 [±6·4]	0·929	0·18 (−1·62; 1·98)	0·845
Sub-scapular skinfold (mm), mean [sd]	17·6 [±6·6]	17·5 [±5·3]	0·921	16·9 [±6·7]	16·3 [±6·5]	0·553	0·66 (−0·94; 2·27)	0·412
Tricipital skinfold (mm), mean [sd]	17·9 [±7·1]	18·3 [±7·0]	0·593	16·8 [±8·0]	16·5 [±7·2]	0·633	0·99 (−0·85; 2·84)	0·286
Abdominal skinfold (mm), mean [sd]	22·0 [±8·0]	21·9 [±9·3]	0·907	21·0 [±9·1]	19·3 [±10·8]	0·149	1·74 (−1·56; 5·04)	0·298
Glucose (mg/dl), mean [sd]	87 [±7]	79 [±8]	**0·000**	88 [±5]	80 [±9]	**0·000**	−0·90 (−4·57; 2·77)	0·627

sd, standard deviation.

* Paired Student's *t* test.

** Linear regression adjusted by the initial value.

## Discussion

This pilot randomised clinical trial demonstrates that two different daily doses of 200 IU and 1000 IU of vitamin D for 4 months increase serum 25(OH)D concentration in a dose-response relationship in healthy young adults. No statistically significant relationship between BMI was found when comparing the intervention group with the control. Lower levels of total cholesterol, LDL-cholesterol and higher levels of HDL-cholesterol were evidenced in the intervention group. However, the decrease was statistically significant only in LDL-cholesterol levels in the intervention group when compared with the control group.

### Vitamin D and BMI

The relationship between vitamin D and BMI is mainly due to the participation of vitamin D as a pre-hormone and its ability to go to many specific tissues in the body^(30)^. Vitamin D is a fat-soluble compound that has been related to obesity due to its deficiency at the serum level in the body^(31)^ due to the sequestration of vitamin D in adipose tissue, which results in lower concentrations of circulating 25(OH)D^(32)^. This hypothesis has been confirmed in a Mendelian randomisation study, which demonstrated a unidirectional causal relationship, specifically, excess fat leads to reduced vitamin D levels^(33)^. The previous is consistent with the results of this pilot study, where no significant change in BMI was found when the treatments were compared. The level of evidence that has been found so far about vitamin D supplementation and excess weight is mainly given in groups that present a base deficiency of vitamin D in the blood, unlike our study, where vitamin D supplementation was in healthy young adults.

### Vitamin D and serum lipids

Various observational studies across the world indicate an association between vitamin D deficiency and low levels of high-density lipoproteins (HDL) and high triacylglycerols, as well as higher levels of apolipoprotein E^(34,35)^. A prospective study evaluating vitamin D levels and blood lipids in vitamin D-deficient adults from the United States showed a significant association between low 25(OH)D levels and hypercholesterolemia^(36)^. Additionally, a systematic review and meta-analysis published in 2019^(4)^ examined the effect of vitamin D supplementation on serum lipid profiles of adults, in randomised controlled clinical trials (RCTs) published before 2018. It concluded that vitamin D supplementation has an effect positive in reducing serum levels of total cholesterol, LDL-cholesterol and triacylglycerols, but not in HDL-cholesterol levels. These findings are consistent with ours, as this controlled trial showed a significant reduction in LDL-cholesterol and a positive effect on triacylglycerol levels in the intervention group. However, for total cholesterol and HDL levels, favourable results were observed for both the group that received 1000 and 200 IU of vitamin D. The dose of vitamin D supplementation has been reported to explain part of the variations between the results in different populations^(22)^. Therefore, observing the impact of different doses within the same population elucidates more specific results and contributes to its better understanding.

One of the strengths of our study was the quantification of the concentration of vitamin D in the blood, which made it possible to follow the intra-person variation in its metabolism. In addition, the analytical reliability of 25(OH)D in the quantification was monitored through DEQAS (the vitamin D External Quality Assessment Scheme), since the laboratory where the quantification was carried out could do it.

Regarding limitations, we did not include a placebo group for ethical reasons. A smaller vitamin D dose of 200 IU/d was included to provide an adequate dose-response comparison with the higher dose. Another limitation is that being a pilot study the sample size was not optimal, and there was no evidence of significance in our findings on total cholesterol, HDL-cholesterol and triacylglycerols.

Despite the lack of solid conclusions about the clinical benefits of vitamin D supplementation in published studies, achieving vitamin D sufficiency remains crucial in patients with cardiovascular and metabolic conditions^(22)^. The implications for public health must be given mainly in the implementation of a public policy of vitamin D supplementation and a regular quantification of this in blood at early ages and the long term.

In conclusion, we found that two different doses of vitamin D supplementation, 200 IU *v*. 1000 IU, produced changes in serum 25(OH)D levels over 15 weeks (approximately 4 months) in healthy young adults. There were no adverse effects of supplementation at any dose. No significant change in BMI was evident when the treatments were compared. However, a significant reduction in LDL-cholesterol was found when the two intervention groups were compared.
